# Cyclodextrin glycosyltransferase immobilization on functionalized graphene nanoplatelets for enhanced cyclodextrin production

**DOI:** 10.1371/journal.pone.0326099

**Published:** 2025-06-26

**Authors:** Babatunde A. Ogunbadejo, Sulaiman Al-Zuhair

**Affiliations:** Department of Chemical and Petroleum Engineering, UAE University, Al Ain, UAE; International Iberian Nanotechnology Laboratory, PORTUGAL

## Abstract

Immobilization of enzyme on functionalized nanomaterials offers a promising strategy for enhancing enzyme stability, reusability, and activity in a variety of industrial applications. In this study, graphene nanoplatelets (GNPs) were functionalized with ethanolamine, 3-aminopropyltrimethoxysilane (APTMS), and 3-mercaptopropyltrimethoxysilane (MPTMS) to provide an efficient support for cyclodextrin glycosyltransferase (CGTase) immobilization. Thermogravimetric analysis (TGA) verified the successful incorporation of functional groups, including amine and thiol groups, and ethanolamine-functionalized GNPs showing the highest grafting levels. The maximum starch conversions observed with the functionalized supports were 21% for APTMS-functionalized GNP, 11% for ethanolamine-functionalized GNP and 7% for MPTMS-functionalized GNP. After 16 hours of continuous operation, ethanolamine- and APTMS-functionalized GNP retained 94% of their initial activity, while MPTMS-functionalized GNP maintained 86% residual activity at the end of the operation. Production rate of cyclodextrin reached 171 mg/g/hr for GNP-Ethanolamine, 185 mg/g/hr for GNP-APTMS and 90 mg/g/hr for GNP-MPTMS per gram of immobilized CGTase. This study showed that functionalized GNP exhibited better catalytic performance and good stability during cyclodextrin synthesis, hence, they demonstrate strong potential for developing commercial biocatalyst, paving the way for further advancements in using GNP-based supports in CGTase immobilization.

## 1. Introduction

The growing interest in biocatalysis stems from its potential to enable more sustainable and efficient chemical processes. Among the many enzymes employed in this field, cyclodextrin glycosyltransferase (CGTase E.C. 2.4.1.19) plays a pivotal role in the production of cyclodextrins (CDs). CGTase acts on α-(1,4) glycosidic bonds, catalyzing four key reactions: cyclization, coupling, disproportionation, and hydrolysis. The cyclization reaction is particularly important, leading to the formation of CDs, which primarily consist of six, seven, and eight glucose monomers, forming α-, β-, and γ-CD, respectively [1–3]. These cyclic oligosaccharides have attracted significant attention due to their ability to form inclusion complexes with various organic and inorganic molecules, enabling their use in a wide range of industries, such as pharmaceuticals, food, cosmetics, agrochemicals, textiles, and more [4].

To enhance CGTase performance for industrial applications, efficient enzyme immobilization is essential as immobilization improves enzyme stability, reusability, and catalytic activity, which ultimately results in reducing operational costs and making processes more sustainable. Various supports have been proposed for CGTase immobilization, including mesoporous silica, hydrogels, and sol-gel matrices [5–8]. However, sol-gels exhibit low immobilization efficiency, and the bulkiness of starch molecules restricts their accessibility into the matrix pores. Additionally, immobilization occurs during sol-gel synthesis, which involves relatively harsh curing conditions that may lead to enzyme activity loss. In hydrogels, immobilized enzymes are prone to leaching as the matrix swells upon contact with water. While mesoporous silica offers a high surface area, making it a promising immobilization material, the presence of surface charges often results in poor enzyme retention and increased enzyme deactivation. Graphene nanoplatelets (GNPs) have emerged as promising carriers for enzyme immobilization due to their high surface area, strong mechanical strength, and excellent chemical and thermal stability [9,10]. GNPs consist of multiple stacked graphene sheets layers, preserving the sp^2^ hybridized carbon network. This structural integrity gives the GNPs their good mechanical, electrical, and thermal properties, making them highly versatile for applications in electronics, energy storage, composites, sensors, biomedical fields, as well as in enzyme immobilization [9]. Also, carbon-based materials like GNP are considered as non-toxic and biocompatible, and it can also be derived from renewable sources, thus, offering a sustainable alternative to other traditional supports [11].

Functionalizing GNPs with surface modifiers can enhance their compatibility with enzymes, facilitating stronger interactions between enzyme molecules and the carrier. For example, amine-functionalized GNPs were used to immobilize laccase from *Aspergillus oryzae* via covalent bonding, resulting 80% of the enzyme’s activity after six cycles of pyrogallol degradation [12]. Similarly, ionic-liquid modified graphene oxide immobilized laccase, resulting in enhanced thermal stability, organic solvent tolerance, and operational efficiency [13]. Despite these successes with other enzymes, CGTase immobilization on functionalized GNPs has not been extensively explored. Prior studies have tested various immobilization strategies including, adsorption, covalent binding, and cross-linking, using both organic and inorganic supports [14]. However, these methods often encounter challenges, such as restricted enzyme mobility, reduced catalytic activity and substrate diffusion limitations. Covalent bonds may also cause unfavorable conformation changes in the enzyme structure such as restricted enzyme mobility, reduced catalytic activity, or substrate diffusion limitations due to conformational changes in the enzyme structure [14,15].

Proper enzymes orientation on the support material is critical to maximizing enzyme stability and minimizing undesirable interactions, especially in reactions involving bulky substrates like starch. For example, thiol functionalization epoxy supports have been shown to facilitate site-directed covalent immobilization of proteins via disulfide bonds [16]. This approach was used for CGTase immobilization on agarose beads, where disulfide linkages were found between thiol groups on the protein surface and reactive groups on the support [17]. However, for effective immobilization, the enzyme must be treated with reducing agents like dithiothreitol (DTT) to release free thiol groups However, for effective immobilization, the enzyme must be treated with reducing agent like dithiothreitol or 2-mercaptoethylamine to release free thiol groups [15].

In this study, use of functionalized GNPs for CGTase immobilization was explored to address the limitations associated with traditional supports. Functional groups were grafted onto GNP by two methods; (1) Silanization in an organic solvent using 3-aminopropyltrimethoxysilane (APTMS) and 3-mercaptopropyltrimethoxysilane (MPTMS) to generate amine-functionalized GNP-NH and thiol-functionalized GNP-SH supports, respectively. GNP-NH, when activated with glutaraldehyde, can covalently bind to the amino-terminal groups on the lysine residue of the CGTase. Whereas GNP-SH enables covalent attachment through disulfide linkage with the native cysteine residue of CGTase. These two approaches are expected to enable site-directed immobilization. (2) Ethanolamine functionalization, wherein the bi-functional chemical structure of ethanolamine, containing both amine and hydroxyl groups, facilitating interaction with both the enzyme and the GNP surface in a variety of ways. The reactive amine groups present on the surface of ethanolamine-functionalized graphene (GNP-NH_2_) would allow covalent binding sites to CGTase. The Immobilized CGTase systems were evaluated on the supports for catalytic performance, including activity, optimum temperature and pH, and stability in continuous packed-bed reactors. Immobilizing CGTase on functionalized GNPs offers several advantages over conventional supports. On one hand, higher loading capacity is due to large surface area of functionalized GNP, enhancing catalytic efficiency. On the other hand, improved enzyme-substrate interactions through targeted functional groups, resulting in enhanced stability. Additionally, the immobilized CGTase on functionalized GNPs system allows for easy recovery and reuse, reducing operational costs and minimizing waste production.

This study not only demonstrates the feasibility of using functionalized GNPs for CGTase immobilization but also highlights the potential of these supports for other biocatalytic applications. The results provide new insights into efficient cyclodextrin production and open avenues for developing advanced enzyme immobilization systems, enabling the biocatalytic production of various high-value chemicals.

## 2. Materials and methods

### 2.1. Materials

Graphene nanoplatelets (GNPs) (size: 3 nm, purity: 99%, 530 m^2^/g) were purchased from Nanografil Nano Technology (Ankara, Turkey), Ethanolamine, methanol, α-, *β*-, *γ*-CD, 3-aminopropyltrimethoxysilane (APTMS), 3-mercaptopropyltrimethoxysilane (MPTMS), glutaraldehyde was obtained from Sigma Aldrich (St Louis, USA). Toruzyme^®^ CGTase (from *Thermoanaerobacter* sp.) was a kind gift from Novozymes A/S (Bagsvaerd, Denmark). were obtained from Sigma-Aldrich (St. Louis, USA). Ethanol was obtained from J.T Baker (Deventer, Netherlands). Starch soluble (extra pure), phenolphthalein, methyl orange, sodium carbonate (Anhydrous, 99.9%) were obtained from Sisco research laboratory (Maharashtra, India). All other chemicals used were of analytical grade and used as purchased.

### 2.2. GNP functionalization

#### 2.2.1. Ethanolamine functionalization.

Initially, the GNPs were activated by vacuum drying (DAIHAN SOV-30, Korea) at 100 °C for 4 h. Subsequently, 500 mg of the activated GNPs were added to a 50% v/v methanol-ethanolamine solution and stirred for 30 mins at 40 °C using a magnetic stirrer (DAIHAN SMHS-3, Korea) to ensure thorough functionalization. The resulting mixture was then centrifuged (OHAUS FC5816, USA) at 2290 × g (relative centrifugal force) for 5 min and dried in an oven at 100 °C for 4 h. The produced functionalized support was designated as GNP-NH_2_. It was further activated by treating with 5 mL of 25% v/v glutaraldehyde solution, yielding a final product labeled as GNP-NH_2_-G.

#### 2.2.2. Functionalization using APTMS and MPTMS.

The GNPs were initially heat-treated in a vacuum oven at 100 °C for 4 h. Subsequently, 200 mg of GNPs were transferred into a round-bottom flask containing 25 mL of ethanol and dispersed via ultrasonication (Q55 Qsonica, USA) for 25 min. Afterward, 1 mmol of either APTMS or MPTMS per gram of GNPs was added to the solution, followed by additional 10 min of ultrasonicated. The mixture was then stirred at 65 °C for 4 h, following the procedure outlines in previous studies [18,19]. After functionalization, the mixture was centrifuged, washed with ethanol, and dried under vacuum at 80 °C for 2 h. The resulting products are referred to as GNP-NH (functionalized with APTES) and GNP-SH (functionalized with MPTMS), respectively. GNP-NH required activation with glutaraldehyde prior to use, while GNP-SH was directly suitable for enzyme immobilization due to its inherent nucleophilicity [20]. To activate GNP-NH, 10 mg of the material was treated with 0.5 mL of 5% (v/v) glutaraldehyde solution prepared in phosphate buffer (pH 6.2) at 25 °C for 2 h. Following the activation, the support (now referred to as GNP-NH-G) was thoroughly rinsed with water to remove any excess reagent, making it ready for subsequent applications.

### 2.3. Enzyme immobilization

A 0.5 mL aliquot of CGTase solution (0.39 mg/mL) was added to 10 mg of each support, namely GNP, GNP-NH2-G, GNP-NH-G, GNP-SH, and incubated overnight at 25°C under continuous stirring at 100 rpm in an incubator (Labtech LSB-015S, KOREA). After incubation, the supports were separated from the mixture by centrifugation and washed with 5 mL of buffer solution to remove non-covalently bound proteins. The concentration of CGTase in the solution was measured before and after incubation following the method described elsewhere [3]. The uptake and immobilization yield were calculated using Eqs (1) and (2), respectively.


$$\begin{array}{c} q_{e}\;=\frac{\left(C_{initial}{\cdot V}_{initial}\right)-\left[\left(C_{final}{\cdot V}_{final}\right)+\left(C_{wash}{\cdot V}_{wash}\right)\right]}{m} \end{array}$$ (1)

$$\begin{array}{c} Immobilization\;yield\;(I.Y)\;=\frac{\left(C_{initial}\right)-\left[\left(C_{final}\right)+\left(C_{wash}\right)\right]}{C_{initial}}x\;100\% \end{array}$$ (2)

### 2.4. Characterization

X-ray diffraction (XRD) analysis was performed using a Cu Kα-equipped X-ray diffractometer (PANalytical Instrument, X’Pert3 Powder, Philips, Holland) operating at 40 mA. The XRD patterns of both empty supports and enzyme-adsorbed samples were recorded with a step size of 0.026°/min across a 2θ range of 5°-80°. Fourier transform infrared spectroscopy (FTIR) (Jasco Corporation FT/IR-6300, Japan) was used to investigate the functional groups on the supports in three conditions: without enzyme, with adsorbed enzyme, and after the enzymatic reaction. The samples were prepared by mixing with potassium bromide (KBr; Sigma-Aldrich) and drying at 105 °C to prevent interference from moisture. The FTIR spectra were recorded within range of 400–4000 cm^−1^. Thermogravimetric analysis (TGA) was conducted on 10 mg of each sample to evaluate their thermal stability and quantity of functional groups present on the GNPs. The analysis was performed over a temperature range of 0 °C–700 °C using a TGA Q50 V20.10 Build 36 analyzer (TA Instruments, Haan, Germany). The heating rate was set at 20 °C/min under continuous nitrogen flow to prevent oxidation during the measurement. The quantity of each introduced functional group was determined through thermal stability analysis using TGA. Most functional groups decompose between 200–400 °C, and the difference in weight loss between pure and functionalized GNPs within this temperature range was used to calculate the molar amount using Eq (3).


$$\begin{array}{c} Molar\;amount\;of\;functional\;group\;(mmol)=\;\frac{diff.\;in\;weight\;loss\;(\%)\times mass\;of\;sample\;(mg)}{Molecular\;weight\;of\;functional\;group\;(\frac{g}{mol})} \end{array}$$ (3)

Where the weight loss (%) is the percentage of initial sample lost during the process, total organic mass lost (mg/g) represents the amount of organic matter lost (mg) per gram of sample used and the organic amount/ g of sample stands for the amount of organic matter lost (mmol) per gram of sample used.

### 2.5. CDs production and operational stability

The CDs production experiment was carried out in a jacketed glass column (internal diameter: 7 mm, length: 100 mm, volume: 3 mL) containing 5 g immobilized CGTase. A starch solution (10 g/L) was pumped from a feed flask, maintained at 25 °C with stirring at 150 rpm, at a flow rate of 1 mL/min using a peristaltic pump (Minipuls 3, Gilson, USA). Product samples were collected at pre-specified intervals and analyzed for CDs concentration using high performance liquid chromatograph (HPLC) equipped with an ultra-amino column (250 x 4.6 mm, 5 μm, Restek) and a refractive index detector (RID-20A, Shimadzu). A mixture of acetonitrile and water (65:35% v/v) was used as the mobile phase at 60 °C with a flow rate of 1 mL/min. The efficiency of the reactor was evaluated based on starch conversion to CDs.

## 3. Results and discussion

### 3.1. Characterization of GNP and its derivatives

**Fig 1:**
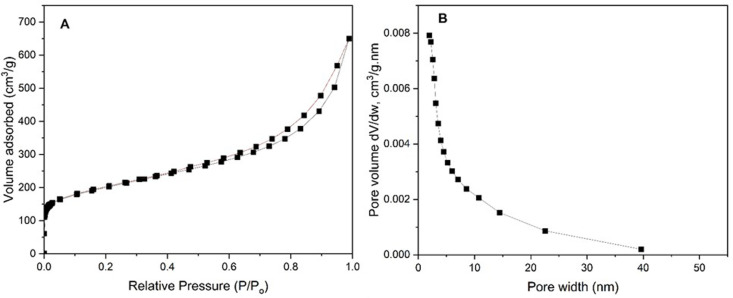
N_2_ adsorption-desorption results of GNP (a) isotherm and (b) pore size distribution.

Fig 1(a) and 1(b) show the N_2_ adsorption-desorption isotherms and pore size distribution of GNPs, respectively. The GNPs displayed a type II isotherm with an H3-like hysteresis loop, characteristic of plate-like particles aggregates. The BET surface area was measured to be 844.2 m^2^/g, with micropore and external surface areas, determined by using the t-plot method, amounting to 345.6 and 498.6 m^2^/g, respectively. The pore size distribution indicates that most pores are smaller than 3 nm, suggesting that CGTase immobilization will likely be limited by the exterior surface of the GNPs. The particle size distribution (PSD) shown in Fig 1 influences the efficiency of enzyme immobilization. CGTase has a relatively large hydrodynamic diameter of 3.6 nm compared to the pore size observed on the GNP. As a result, the majority of CGTase immobilization occurs on the surface of the GNP. By attaching most of the CGTase to the surface, internal diffusion resistance is minimized, leading to improved catalytic performance. However, thermal stress may induce molecular vibrations that allow some CGTase molecules to enter the pores, which could have a positive effect on enzyme retention.

**Fig 2:**
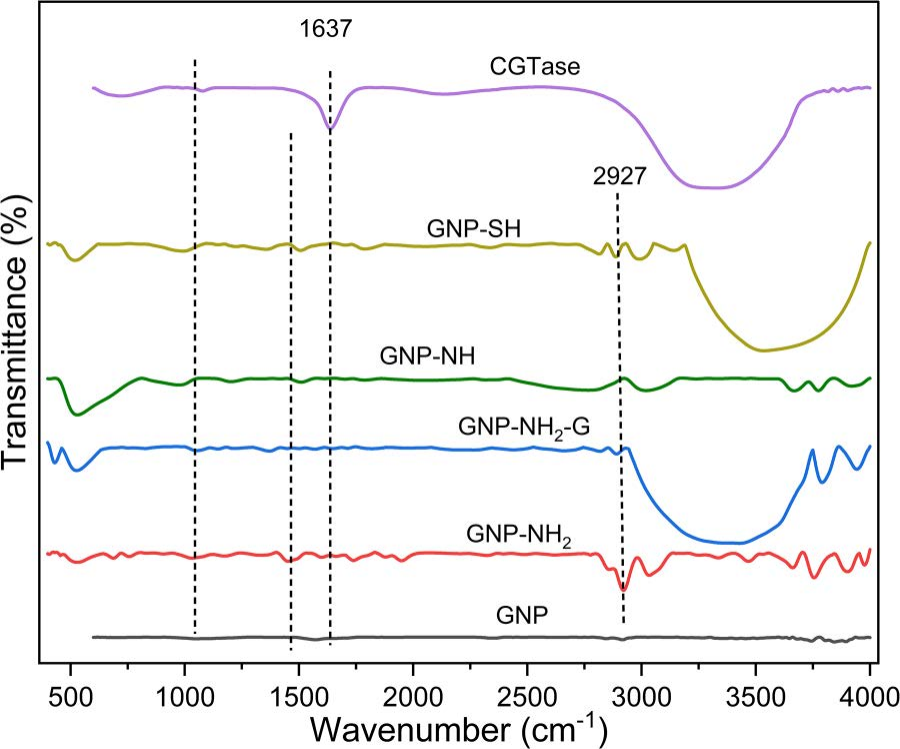
FTIR Spectra of GNP, its derivatives and CGTase.

Fig 2 presents the FTIR spectra of GNP, GNP-NH_2_, GNP-NH_2_-G, GNP-NH, GNP-SH and free CGTase, providing insight into the interaction between functionalized GNP and CGTase. The CGTase enzyme exhibits characteristic peaks at 1640 cm^−1^ and 1045 cm^−1^, corresponding to the N-H bond in the amide I region and the C–O–C bond in glycosylated residues, respectively [21]. In the GNP spectrum, the peaks around 3400 cm^−1^ are attributed to presence of hydroxyl groups, the peak at 2927 cm^−1^ shows the symmetric C-H vibration, the peak at 2343 cm^−1^ represents asymmetric C–H vibration, and the peaks at 1565 and 1130 cm^−1^ represents C=C stretching, and C-O stretching vibrations respectively [22–24]. Additionally, a band at 1620 cm^−1^ corresponds to the sp^2^ framework of graphene. After functionalizing GNP with the amine-containing agents, additional peaks were observed at 1570 and 1370 cm^−1^ representing the N-H bending vibration of primary amines and C-N stretching [22]. Following the treatment with glutaraldehyde, peaks above 3000 cm^−1^ became broader, which indicates the introduction of H-bonding (O-H) from the glutaraldehyde, confirming the activation of GNP-NH_2_. For MPTMS-functionalized GNP, peaks at 2925 cm^−1^ and 2864 cm^−1^ correspond to C–H stretching from CH₃ and CH₂ groups in the propyl spacer, respectively. The band at 1160 cm^−1^ indicates Si-O-C stretching, confirming the synthesis of GNP-SH. Furthermore, the presence of functionalized –SH groups on the GNP surface is evidenced by the –SH stretching vibration at 2576 cm^−1^ [18].

### 3.2. GNP functionalization

**Fig 3:**
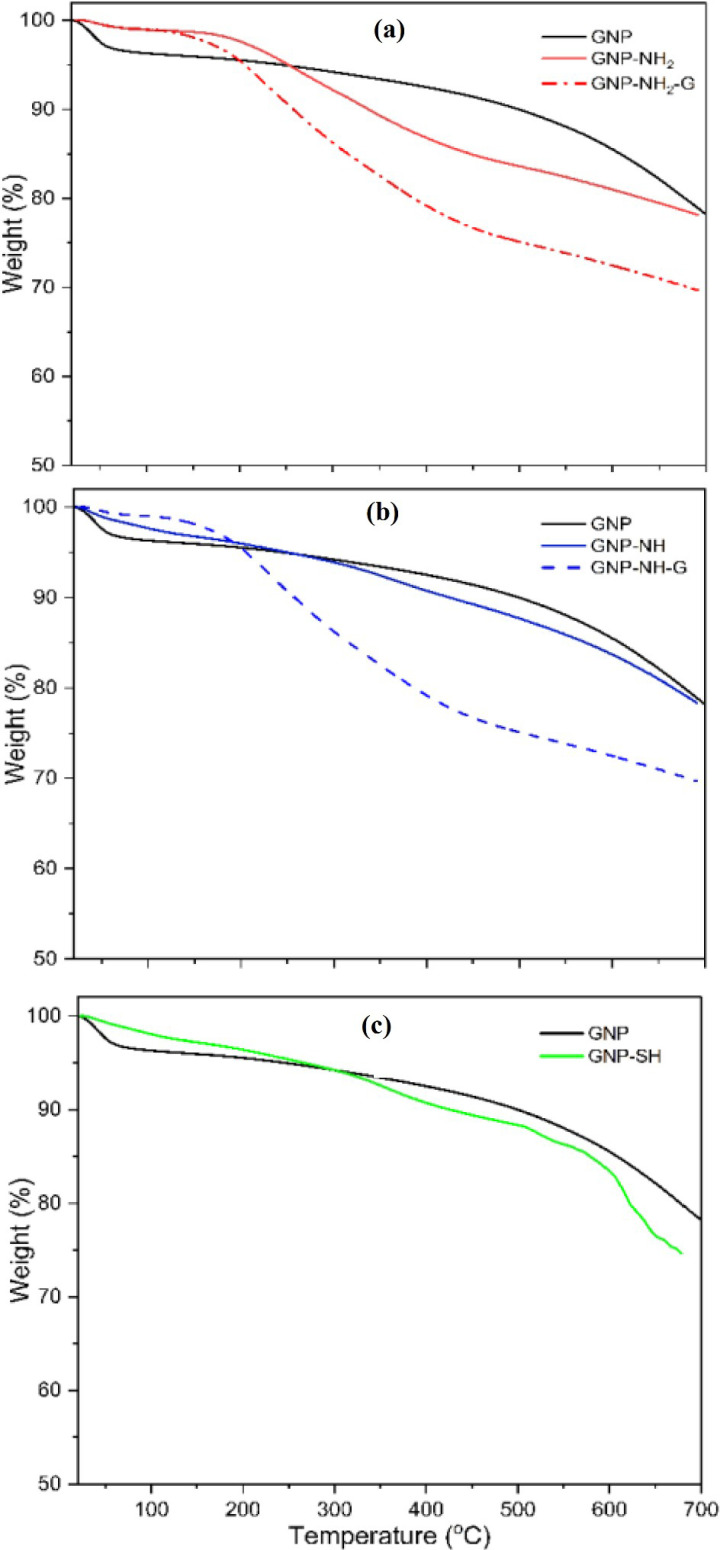
Thermal stability studies for functional group quantification using (a) ethanolamine (b) APTMS (c) MPTMS.

Following the introduction of functional groups into GNP using ethanolamine, APTMS and MPTMS, the thermal stability results are presented in Fig 3. The thermograms of GNP, functionalized GNP, and glutaraldehyde activated GNP showed that these materials exhibit high thermal stability, with minimal structural damage or weight loss within the temperature range below 100 °C, well within the range suitable for enzymatic reactions. The slight weight loss observed below 150 °C is attributed to the evaporation of adsorbed water molecules. The primary degradation occurred between 150 and 650 °C, resulting from decomposition of organic components introduced through the added functional groups.

Table 1 shows the percentage of weight loss across different temperature ranges and the number of functional groups attached in each functionalization step. Ethanolamine introduces both hydroxyl hydroxyl (-OH) and amine (-NH_2_) groups to the GNP surface. 19.2% weight loss observed between 150°C to 650°C is attributed to the decomposition of ethanolamine and the volatilization of hydroxyl and amine groups. Similarly, APTMS adds amine (-NH_2_) groups by forming covalent bonds through the alkoxysilane moiety. The APTMS-functionalized GNP exhibited a 15.8% weight loss, corresponding to the degradation of the silane framework and amine groups. MPTMS, on the other hand, grafts reactive thiol (-SH) group onto GNP surface, which are advantageous for bio-catalytic applications. The MPTMS-functionalized GNP displayed a 20.7% weight loss within the same temperature range. As shown in Table 1, the amount of amine groups introduced using ethanolamine (3.41 mmol) was higher than that achieved with APTMS (1.28 mmol), suggesting that ethanolamine forms a denser amine layer, making it more suitable for covalent bonding with CGTase. Additionally, the thiol content introduced with MPTMS was measured at 2.11 mmol, confirming the successful grafting of functional groups onto the GNP surface.

**Table 1:** Percentages of weight losses of different supports in different temperature ranges.

Sample	Weight loss (%)		Total organic mass lost (mg/g)^1^	Organic amount/ g of sample
	<150 °C	150–650 °C		
GNP	4.07	13.75	137.5	–
Ethanolamine				
GNP-NH_2_	1.3	19.2	192.0	3.41 mmol amino
GNP-NH_2_-G	2.0	27.0	270.0	0.78 mmol glutaraldehyde
APTMS				
GNP	4.07	13.75	137.5	–
GNP-NH	3.2	15.8	158.0	1.28 mmol amino
GNP-NH-G	2	27.0	270.0	1.12 mmol glutaraldehyde
MPTMS				
GNP	4.07	13.75	137.5	–
GNP-SH	2.8	20.7	207.0	2.11 mmol mercapto

^1^Determined from the TGA result from 150–650 °C.

### 3.3. CD production using immobilized CGTase

CGTase was immobilized on GNP and its derivatives functionalized using ethanolamine, APTMS-glutaraldehyde, and MPTMS. To prevent protein crowding, a moderate enzyme concentration of 0.4 mg/mL was used [15]. In preliminary tests, the activity of free CGTase was measured at room temperature for up to 24 h. At the end, CGTase maintained good activity, confirming that the immobilization conditions did not impair CGTase activity [10]. Using the same protein concentrations, high CGTase uptakes were observed on the supports: 17.7 mg/g (± 0.02) for GNP, 18.95 mg/g (± 0.03) for ethanolamine-functionalized GNP, 19.36 mg/g (± 0.04) for APTMS-functionalized GNP and 18.95 mg/g (± 0.04) for MPTMS-functionalized GNP, corresponding to immobilization yields of 90.0%, 97.3%, 99.38% and 97.30%, respectively. The CGTase uptake for the functionalized GNP showed better performance than some of the reported works in the literature, for example, CGTase immobilized on Eupergit C gave 8.1 mg/g [25], 4.1 mg/g was reported over activated silica X030 [26], and 0.73 mg/g was noted using controlled pore silica [7]. Although, a comparative uptake of 21 mg/g was reported over material synthesized from calcium and trimesic acid [10]. These results indicate that the grafting of functional groups onto GNP significantly enhanced its properties for CGTase immobilization, with APTMS-functionalized GNP showing the highest CGTase uptake, likely due to an increase in the number of amine groups on its surface, as verified by TGA results. Immobilization via the cysteine residue (GNP-SH) yielded slightly lower CGTase uptake and efficiency than immobilization through lysine residue, potentially due to the limited SH groups in CGTase from *Thermoanaerobacter sp.* (source of Toruzyme used), which has only one Cys residue per mol of enzyme [15,27]. This result aligns with previous findings of CGTase immobilization on functionalized silica supports [15], where better access to amino groups appeared to enhance immobilization efficiency over that of Cys residues.

**Fig 4:**
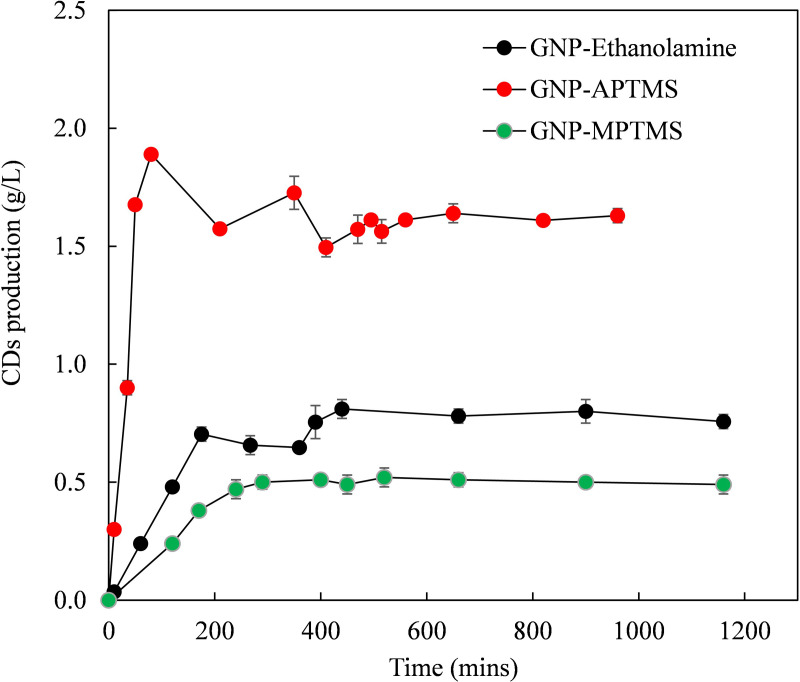
CD production using immobilized CGTase on GNP derivatives.

Soluble starch (10 g/L) used as the substrate in a continuous packed-bed reactor to assess CDs production and operational stability of immobilized enzymes. Total CDs production was calculated by summing the quantities of α-CD, β-CD, γ-CD in the product, as shown in Fig 4. Using 5 g of immobilized CGTase per reactor, maximum starch conversions were achieved as follows: 11% for ethanolamine-functionalized GNP, 21% for APTMS-functionalized GNP, and 7% for MPTMS-functionalized GNP. CD productivities per gram of immobilized CGTase were 171 mg/g/hr for GNP-Ethanolamine), 185 mg/g/hr for GNP-APTMS) and 90 mg/g/hr for GNP-MPTMS. Initial CDs concentrations were 1.14 g/L for GNP-NH_2_-G-CGTase (Ethanolamine), 3.77 g/L for GNP-NH-G-CGTase (APTMS) and 0.25 g/L for GNP-SH. These findings highlight that the nature and type of functional group grafted onto GNP significantly influence its performance as an immobilization support. Functional groups affect the chemical bonding with the GNPs surface, altering surface characteristics such as reactive sites, charge and hydrophilicity, which are the key factors that impact CGTase binding efficiency and activity. The ethanolamine-functionalized GNP with the highest functional groups didn’t translate to better performance probably due to alterations in the CGTase structure post-immobilization [28]. In a related study using silica functionalized by APTMS (Si-NH) and MPTMS (Si-SH), starch conversion reached 22% with Si-NH, yielding 8.71 mg/mL of CDs at a CD production rate of 93.31 mg/g/hr, with an initial starch concentration of 4% (w/v), while Si-SH achieved a 14% conversion, producing 5.69 mg/mL of CDs at a rate of 73.32 mg/g/hr [15]. Comparing the CD production rates using similar functionalization route for silica and GNP, the rate obtained in this work via APTMS-functionalization (185 mg/g/hr) showed close to 100% increment over that reported for silica (93.31 mg/g/hr). Also, the rate for MPTMS-functionalized GNP (90 mg/g/hr) was higher compared to that over silica.

In another study using CGTase immobilized on Amberite IRA 900 and crosslinked enzyme aggregates (CLEA) of CGTase, the CD productivity observed were 7.2 and 84 mg/g/hr respectively [29,30]. Comparing these results to those obtained in the present work show that the higher CD production rate over functionalized GNP could position GNP as a great support for CGTase.

Correlating the performance of each of the support to the thermal degradation results in section 3.2, GNP functionalized with APTMS which exhibited the smallest loss of amine groups compared to ethanolamine-functionalized GNP under similar conditions demonstrated higher catalytic performance. Thus, as the loss of functional groups during the TGA analysis increases, CD production will likely decrease as their will be less attachment of CGTase to the functional groups.

In terms of CDs production stability, GNP-NH_2_-G-CGTase retained 94% of its initial activity (based on maximum CDs production) after 16 h of continuous operation, GNP-APTMS maintained 86%, and GNP-SH retained 94% by the end of the operation. The results indicated that attachment of CGTase to supports via suitable functional groups enhances the reusability of CGTase. CDs yields were higher with amino-functionalized derivatives than thiol-functionalized derivatives.

These results presented in this study showed how GNP could be effectively utilized in CGTase immobilization. The successful functionalization of GNP, which was confirmed by TGA results enhanced the CGTase-GNP interaction, leading to good uptake of CGTase observed on the supports. Using the immobilization strategy of introduction favourable functional groups to the support surface before CGTase immobilization ensures that the enzymatic activity of CGTase is maintained and extends the enzyme operational lifetime. Some of the limitations in this work include the low starch conversion, which could be improved by pre-hydrolysis of the starch into shorter chains before use in CD production. Future works on the use of functionalized GNP for CD production would include the use of membrane bioreactors instead of packed bed reactor for selective removal of cyclodextrin and improving reactant conversion. Also, a parametric study for optimizing all the applicable paramet3ers in the CD production using functionalized GNP needs to be investigated.

## 4. Conclusions

In this study, graphene nanoplatelets (GNPs) were successfully functionalized with ethanolamine, 3-aminopropyltrimethoxysilane (APTMS), and 3-mercaptopropyltrimethoxysilane (MPTMS) to serve as effective supports for cyclodextrin glycosyltransferase (CGTase) immobilization. Thermogravimetric analysis (TGA) confirmed the introduction of amine and thiol functional groups, revealing significant weight losses corresponding to the breakdown of grafted groups. Ethanolamine-functionalized GNPs demonstrated the highest degree of grafting, highlighting their suitability for enzyme immobilization. CGTase immobilization of these functionalized GNPs resulted in enhanced enzyme stability and reusability, which are key factors for industrial applications. The GNPs’ large surface area, combined with the amino and thiol groups from functionalization, provided a robust biocatalytic platform for cyclodextrin synthesis. In biocatalysis, where enzyme stability and activity are vital for process efficiency, this method provides a promising approach for effective enzyme immobilization. Future studies should focus on optimizing immobilization conditions on the functionalized GNP, evaluating the long-term operational stability of the immobilized enzyme, and exploring the scalability of this approach for large scale cyclodextrin production. Functionalized GNPs offer a versatile platform that can be immobilizing various enzymes and supporting diverse biocatalytic processes, paving the way for new applications in biotechnology and industrial development.

## Supporting information

S1 Data Raw Experimental data for CGTase immobilization on GNP. (XLSX)
